# Lead sulphide colloidal quantum dots for room temperature NO_2_ gas sensors

**DOI:** 10.1038/s41598-020-69478-x

**Published:** 2020-07-28

**Authors:** Federica Mitri, Andrea De Iacovo, Massimiliano De Luca, Alessandro Pecora, Lorenzo Colace

**Affiliations:** 1 Department of Engineering, University Roma Tre, Via Vito Volterra 62, 00146 Rome, Italy; 2 Institute of Marine Engineering (INM), CNR, Via Fosso del Cavaliere 100, 00133 Rome, Italy; 3 Institute for Microelectronics and Microsystems (IMM), CNR, Via Fosso del Cavaliere 100, 00133 Rome, Italy

**Keywords:** Electrical and electronic engineering, Electronic devices, Quantum dots

## Abstract

Colloidal quantum dots (CQDs) have been recently investigated as promising building blocks for low-cost and high-performance gas sensors due to their large effective surface-to-volume ratio and their suitability for versatile functionalization through surface chemistry. In this work we report on lead sulphide CQDs based sensors for room temperature NO_2_ detection. The sensor response has been measured for different pollutant gases including NO_2_, CH_4_, CO and CO_2_ and for different concentrations in the 2.8–100 ppm range. For the first time, the influence of the QDs film thickness on the sensor response has been investigated and optimized. Upon 30 ppm NO_2_ release, the best room temperature gas response is about 14 Ω/Ω, with response and recovery time of 12 s and 26 min, respectively. A detection limit of about 0.15 ppb was estimated from the slope of the sensor response and its electric noise. The gas sensors exhibit high sensitivity to NO_2_, remarkable selectivity, repeatability and full recovery after exposure.

## Introduction

Nitrogen dioxide (NO_2_) is an important air pollutant since it contributes to the formation of the photochemical smog, the acid rains and it is also central to the formation of fine particles (PM) as well as affecting tropospheric ozone^1,2^.

Besides natural sources (volcanoes, oceans, biological decay, forest fires), a significant amount of NO_2_ results from human activities such as combustion of fossil fuels welding, explosives, refining of petrol and metals, commercial manufacturing, and food manufacturing^3^. These activities can generate high local concentration of NO_2_ that produces significant impacts on human health, especially breathing problems since NO_2_ inflames the lining of the lungs thus reducing immunity to lung infections^4,5^.

Therefore, reliable detection of NO_2_, even at low concentration, is critical in many different sectors such as industrial plants, indoor and outdoor air quality assurance, automotive and so on, and it is an enabling technology for automatic systems capable of taking necessary measures to reduce unsafe concentrations through ventilation, filtering or catalytic breakdown^6^.

At present, accurate measurement of air quality is possible only by means of large monitoring equipment or laboratory instruments, unsuitable for pervasive monitoring purposes. Alternative low-cost, small-size, ultra-low-power gas sensor platforms have been developed during the last two decades, mainly in the form of components for experimental sensor networks, with promising superior performance in terms of sensitivity, selectivity, minimal drift, fast time response, compactness and low energy consumption^7,8^.

In particular, systems that meet the following characteristics would be of great interest: miniaturization and compatibility with the development of sensor arrays integration with temperature and humidity sensors, simple production techniques and process transferability towards innovative printing techniques.

Today, commercially available gas sensors are mainly based on nanostructured metal oxides (MOx). Such sensors exploit the large surface-to-volume ratio and the high reactivity of the semiconductor material to create chemiresistors whose electrical resistance is related to the target gas concentration^9^. However, these sensors are not selective and need to operate at high temperatures, resulting in significant energy consumption and making their applications unsuitable in several environments^10,11^.

A promising alternative can be provided by Colloidal Quantum Dots (CQDs), an innovative class of nanomaterials widely used in optoelectronics for the realization of photodetectors^12^, environmental sensors^13^ and light-emitting devices, such as LEDs or photoluminescent elements^14^. CQDs are semiconductor nanoparticles suspended in the solution phase that show strong quantum confinement effects, providing unique electronic and optical properties such as increased absorption and emission as well as quantum-size tunability^15^. QDs have an extremely large surface-to-volume ratio hence showing high reactivity with several chemical species even at room temperature; such peculiar chemical characteristics allow a facile doping and functionalization of the QDs even after material synthesis and directly on the deposition substrate^16^. These outstanding characteristics suggests that QDs based sensors can reach high detection performance even if the typical noise current of such devices is quite high, due to the intrinsic granularity of the QD film^17^. The excellent processability of the CQDs enables the use of a wide variety of substrates making them suitable for facile integration on silicon platforms and offering many degrees of freedom in sensor design^18,19^.

Liu et al. reported for the first time a PbS QDs based resistive type gas sensor for NO_2_ detection. The device operated at room temperature with a linear response in the 0.5–50 ppm NO_2_ concentration range and a theoretical detection limit of 84 ppb^20^. Depending on the specific surface chemistry, PbS QDs have been employed also for detection of other gases, such as H_2_S^21,22^, CH_4_^23,24^ and NH_3_^25^.

In addition to PbS, other materials (chalcogenides and MOx) have also been investigated for the realization of gas sensors demonstrating the possibility of a facile fabrication of room temperature chemiresistors for pollutant and hazardous gases. ZnO QDs based sensors were found to be sensitive toward low concentration of NO_2_^26^ and H_2_S^27^. Huang et al. reported a ZnO QDs-decorated graphene nanocomposite with formaldehyde sensing properties^28^. The applicability of SnO_2_ QDs based sensors to NO_2_^10^, H_2_S^29^, ethanol^30^ and LPG^31,32^ detection has been demonstrated as well. Other relevant examples are a graphene QDs based sensor developed for NH_3_ sensing^33^, PbSe QDs sensitive and selective to NO_2_^34^ and WO_3_ QDs based sensor to detect H_2_S gas^35^.

Despite the successful demonstration of several QDs based gas sensors, their performance has never been related to the geometrical characteristics of the proposed devices.

Here we investigate chemiresistive gas sensors for room temperature NO_2_ detection fabricated drop-casting PbS CQDs onto interdigitated metal contacts on silicon and tuning the film thickness.

Beyond the type of sensing material, morphology and temperature, thickness is an important design parameter. Most researchers have used a specific film thickness and very little attention has been devoted to the study of the effect of the film thickness on the gas sensing response.

Here we systematically investigate the influence of the PbS QDs film thickness on the sensor response in order to maximize its performance.

## Experimental section

### Device fabrication

**Figure 1:**
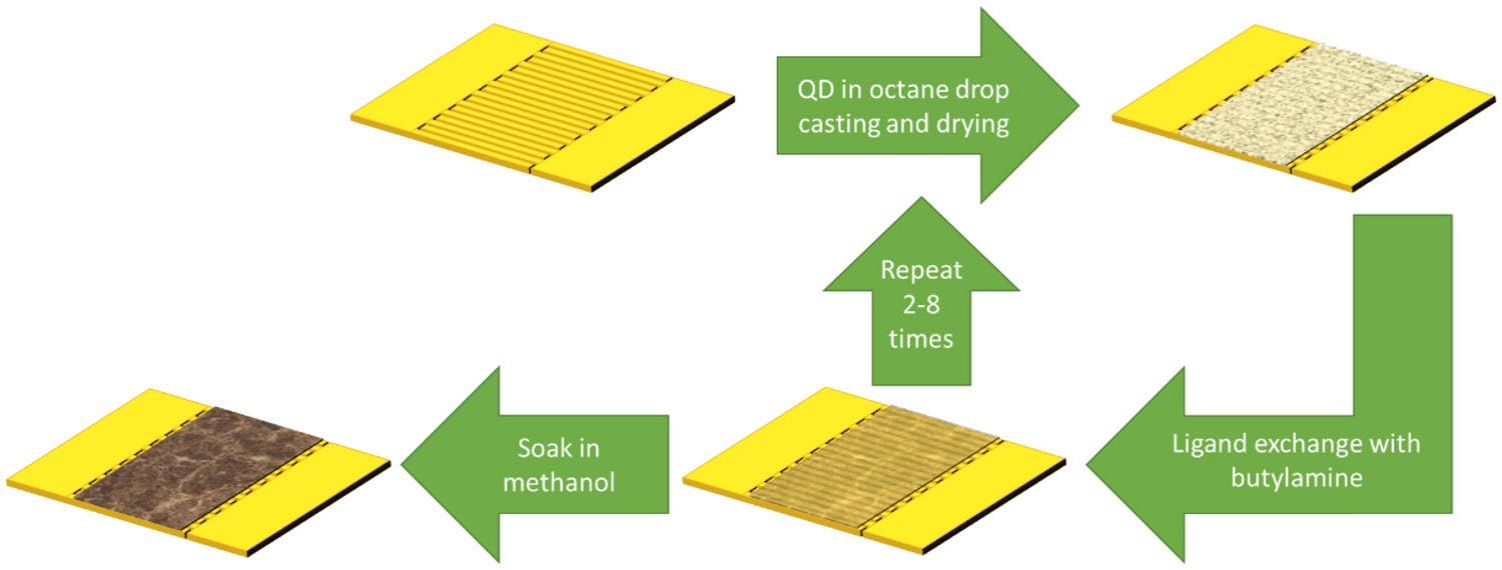
Device fabrication process.

Devices have been fabricated starting from commercial PbS CQDs capped with long-chain oleic acid (OA) and dispersed in toluene (*Sigma-Aldrich 747076*). The mean diameter of the QDs is ~ 4 nm, as confirmed by optical absorption data provided by the producer. We optimized the device fabrication process aiming at the maximization of the QDs surface available for reaction with the analyte gases; at the same time, neighboring QDs in the deposited film should be close enough to allow charge transport. Devices fabrication was carried out to achieve a satisfactory balance between these two needs. OA is essential to stabilize the colloid avoiding agglomeration but, because of its long carbon chains, it hinders efficient gas adsorption and carrier transport, thus a ligand exchange procedure is needed to get rid of the long-chained OA and substitute it with a shorter ligand. To this extent, the QDs dispersion was mixed in excess methanol to remove the organic capping. Nanoparticles precipitation was obtained by centrifugation at 10,000 rpm for 8 min. Excess solvent was dried in vacuum for 24 h. Then, the precipitated and isolated QDs were redispersed in octane to obtain a concentration of about 0.8 mg/mL. The film was deposited using layer-by-layer dropcasting technique onto pre-cleaned interdigitated gold electrodes (IDE) on an oxide-coated silicon substrate. Figure 1 schematically shows the deposition procedure. Briefly, 15 µl of PbS QDs in octane were dropped onto the substrate and the solvent was evaporated in vacuum. Then 15 µl of butylamine was used as a short ligand to redistribute the nanoparticles on the substrate. Residual butylamine was vacuum dried. The film deposition and the ligand exchange treatment processes were repeated for 2 to 8 times, thus obtaining devices with different QDs film thickness in the range from 500 nm to 1.5 µm. Finally, the device was soaked in absolute methanol for 2 h to remove the butylamine in order to reduce the mean distance and to improve the conductivity between the QDs^36^. Moreover, during this last step, the QDs surface was exposed, enabling its reaction with analyte gases. The fabrication process is summarized in Fig. 1.

In this work, we realized devices with a different number of QDs layers to investigate the influence of this parameter on the sensor response. Several devices (more than 40) were realized to account for statistical variation of the sensors’ performance. Due to difficulty in getting precise layer thickness measurements, we will refer to the devices with respect to the number of deposited QDs layers.

### Device characterization

The gas sensing characteristics of the proposed devices were measured in a custom-made 4.5 mL chamber realized in Polyoxymethylene (Delrin). A Keithley 2400 source meter unit was used to continuously measure the sensor resistance during target gas exposure and release.

The gas response measurements were performed at room temperature and 35% controlled humidity with target gas concentration between 2.8 and 100 ppm. The response of PbS QDs based sensors to NO_2_, CH_4_, CO and CO_2_ has been studied.

Gases were injected into the testing chamber through three digital mass flow controllers with a maximum range of 200 sccm each. The desired gas concentration was obtained by diluting the gas with dry nitrogen through the flow controllers. A proportion between the injected flow and the concentration of the gas in the tank was used to determine the gas concentration in the chamber, as described by Eq. 1

1 $$C_{G} = C_{T} \frac{{V_{GT} }}{{V_{GT} + V_{{N_{2} }} }}$$

where C_G_ is the target gas concentration in the gas chamber, C_T_ is the target gas concentration in the gas tank, V_GT_ is the volumetric flow rate of the target gas and V_N2_ is the volumetric flow of N_2_.

A typical test cycle consisted in the following steps: first, the chamber was filled with dry nitrogen until the sensor resistance became stable. Then, the target gas, diluted in nitrogen to reach the desired concentration, was injected into the chamber. After 2 min, dry nitrogen was used to purge the chamber.

The most important device parameter is the gas response (S), defined as the ratio between the sensor resistance measured in dry nitrogen (R_n_) and that in the target gas (R_g_) (Eq. 2)

2 $${\text{S}} = \frac{{R_{n} { }}}{{R_{g} }}$$

We also evaluated the sensor response time (T90) defined as the time needed for the sensor resistance to achieve 90% of the total resistance change upon exposure to the target gas, according to:

3 $$T90 \to R_{{max}} - 90\% \Delta R$$

Similarly, the recovery time (T10) has been evaluated as the time needed for the sensor resistance to reach 10% of the total resistance change after purging the measurement chamber with N_2_, according to:

4 $$T10 \to R_{{min}} + 90\% \,\Delta R$$

## Results and discussion

**Figure 2:**
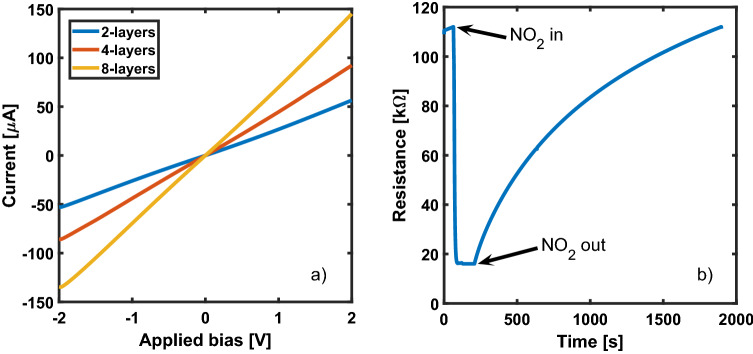
(**a**) Current–voltage characteristics in air atmosphere. (**b**) Real-time resistance change to 30 ppm NO_2_ at room temperature.

QDs based sensors take advantage from the extremely large surface-to-volume ratio and the high reactivity of the semiconductor material to produce chemiresistors whose resistance is related to the concentration of the target gas. In order to work properly, the devices must show a linear current–voltage characteristic, thus implying the formation of an ohmic contact between the QDs film and the metal layer. In Fig. 2a the current–voltage characteristics of three devices with different film thickness are shown; devices are kept in air atmosphere. Linear and symmetrical characteristics are observed, as expected for PbS QDs deposited onto high work function metals, such as Au, that are known to provide ohmic contacts^37^. The typical measured current I at 2 V in air is about 56 µA for 2-layers device, 92 µA for 4-layers device and 145 µA for 8-layers device, with a corresponding resistance of 36 kΩ, 22 kΩ and 14 kΩ, respectively. If characterized in nitrogen atmosphere, the devices still show a purely linear current–voltage characteristic but with a much higher resistance value (110 kΩ, for the 4-layer device). This is expected given the doping effect of oxygen adsorbed on the surface of the QDs^20^.

As expected, there is an inverse proportionality relationship between the measured resistance and the number of deposited QDs layers.

Figure 2b shows the real-time resistance change of a 4-layers PbS QDs gas sensor in response to a 30 ppm NO_2_ flow at room temperature. Initially, the sensor shows a relatively stable resistance of about 110 kΩ.

As soon as NO_2_ is introduced into the testing chamber, the resistance rapidly decreases reaching a minimum value of 16 kΩ. After about 80 s the device resistance reaches a saturation plateau. The NO_2_ exposure lasts two minutes, then the target gas supply is interrupted and N_2_ is flowed to purge the chamber. The sensor resistance gradually returns to its original value, pointing out the capability of full baseline recovery after less than 30 min.

**Figure 3:**
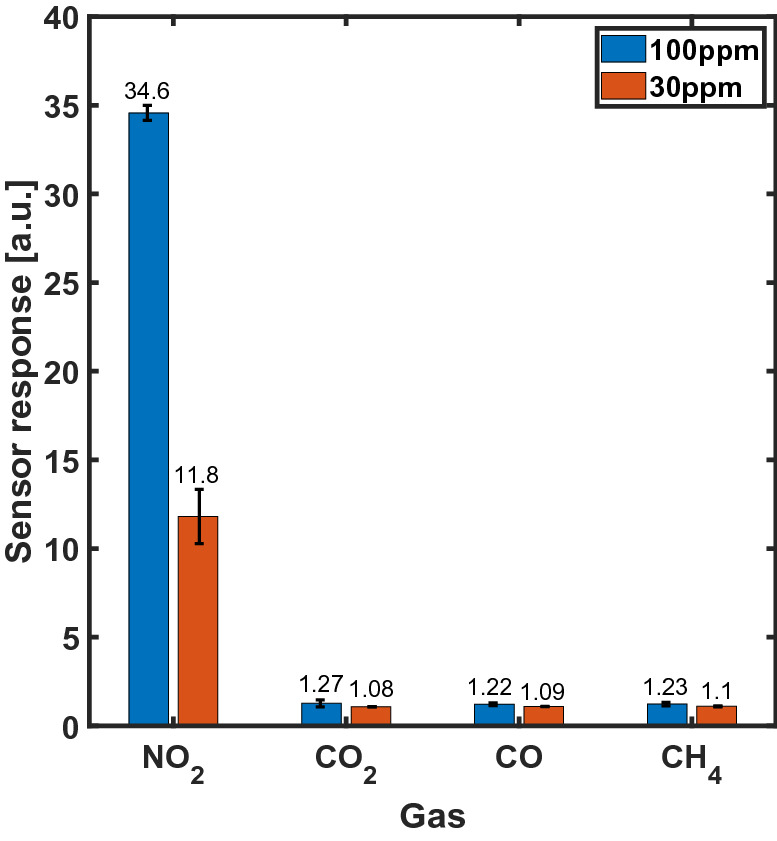
Selectivity of 4-layers sensors based on PbS quantum dots to 100 and 30 ppm NO_2_, CO_2_, CO, CH_4_ at room temperature.

We attribute the sensor response to NO_2_ to the oxidizing effect of the target gas. PbS QDs are well known to be easily p-type doped by the action of oxygen atoms, that introduce deep energy levels in the valence band^20^. NO_2_ has a high oxidation potential, thus it should also act as a p-type dopant for PbS, increasing the number of free holes, hence reducing the device resistivity. To verify this hypothesis, we tested our devices with different gas species, including non-oxidizing gases. As expected, the sensors responded only to NO_2_ flow, showing a high selectivity and negligible response to CO, CO_2_ and CH_4_. Figure 3 shows the measured sensor response for different target gases at 30 ppm and 100 ppm.

**Figure 4:**
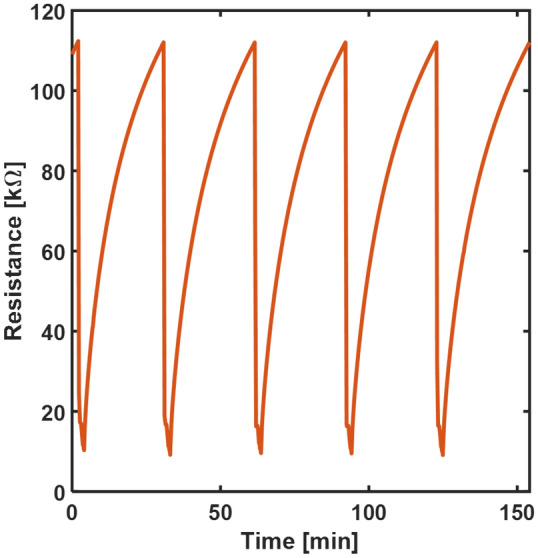
Repetitive cycles of resistance variation towards 30 ppm NO_2_.

We also evaluated the stability of the proposed gas sensor and repeatability of successive measurements. To this extent, we repeated multiple gas flow/purge cycles monitoring the sensor’s resistance. As seen in Fig. 4, the 2-layers sensor shows repeatable performance upon multiple exposures to 30 ppm of NO_2_.

**Figure 5:**
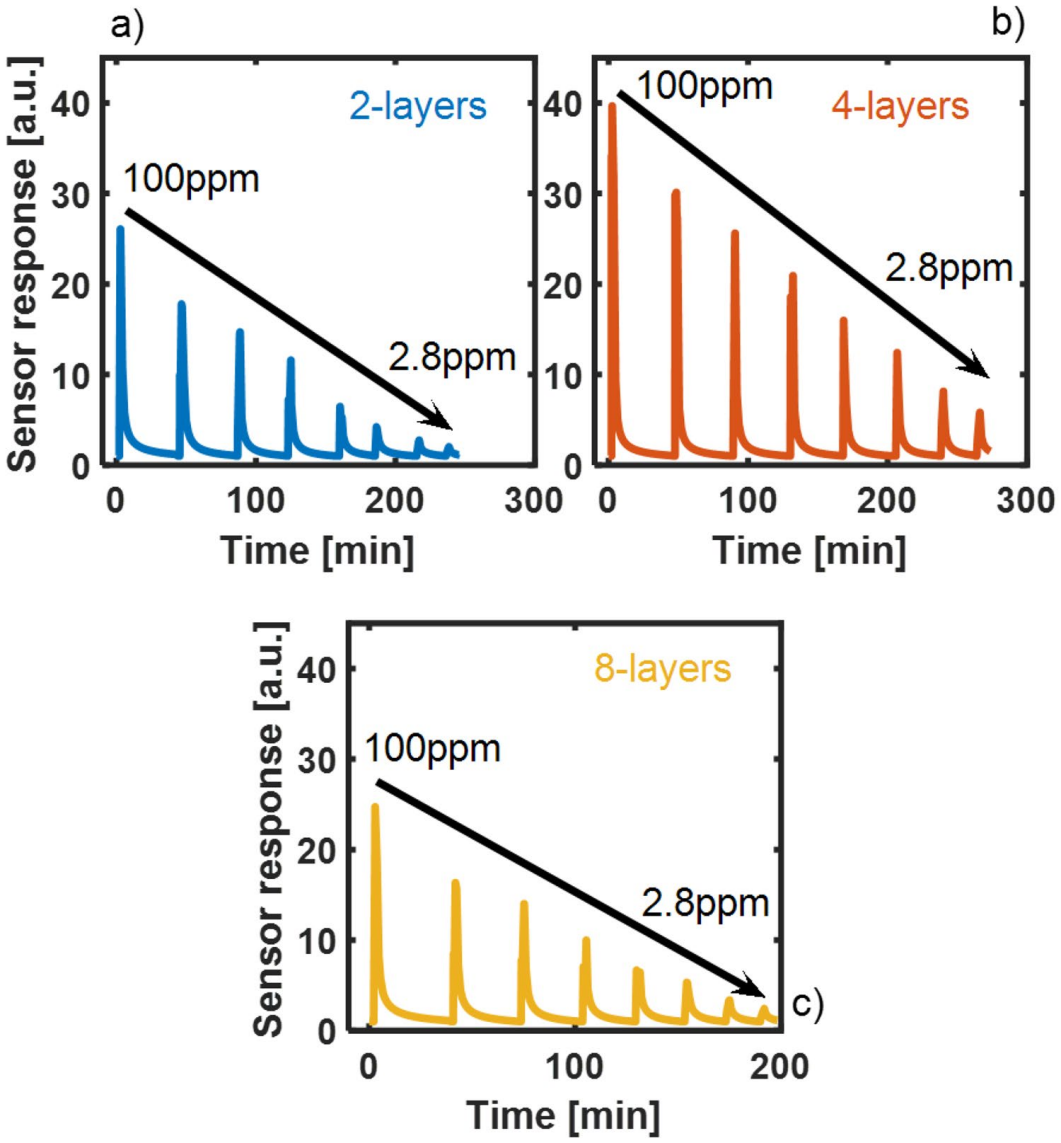
Response curves toward different concentrations of NO_2_ at room temperature for the fabricated (**a**) 2-layers, (**b**) 4-layers and (**c**) 8-layers devices.

In order to optimize the device performance, sensors with different film thickness were tested. In particular, we observed the response curve toward different concentrations of NO_2_ gas at room temperature for 2-layers (Fig. 5a), 4-layers (Fig. 5b) and 8-layers (Fig. 5c) devices. For each device, eight cycles of gas exposure and purge were successively recorded, corresponding to NO_2_ decreasing gas concentrations of 100, 70, 50, 30, 20, 10, 5, 2.8 ppm.

All devices show excellent reversibility and a negligible baseline drift: neither illumination nor thermal treatment was employed to recover the sensor to baseline, unlike what requested by the metal oxide semiconductors (MOx) based resistive-type gas sensors.

A direct proportionality between the sensor response and the NO_2_ gas concentration has been observed in the whole 2.8–100 ppm range, regardless of the film thickness.

All measurements were carried out at a constant relative humidity (35%); water vapor is expected to be an interferent for gas measurements. Preliminary tests showed a negligible variation of the sensor response in the 10–50% humidity range, but further analysis is needed to determine the performance variation for different relative humidity and ambient temperature.

**Figure 6:**
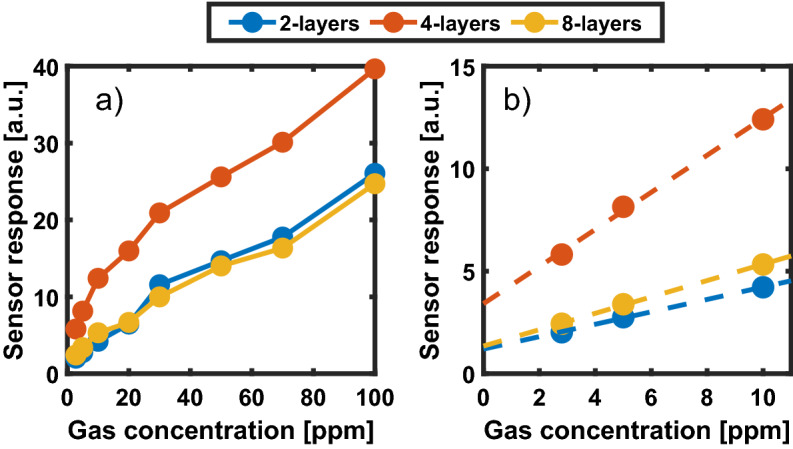
(**a**) Comparison between the responses of the 2-,4- and 8-layers devices at the various concentrations. (**b**) Linear fitting in the 2.8–10 ppm range.

Figure 6a shows the sensor response versus target gas concentration for the 2- 4- and 8-layers devices. The response of the 4-layers sensor is larger in the whole measurement range, and it reaches a value of 40 Ω/Ω at a 100 ppm concentration, whereas the 2- and 8-layers devices only reach a response of 25 Ω/Ω and 26 Ω/Ω, respectively.

This difference is more evident at low gas concentrations: a linear fit of the sensor response versus the NO_2_ concentration in the 2.8–10 range ppm is shown for all devices in Fig. 6b. The slope of the 4-layers device response characteristic is almost three times that of the other two.

**Figure 7:**
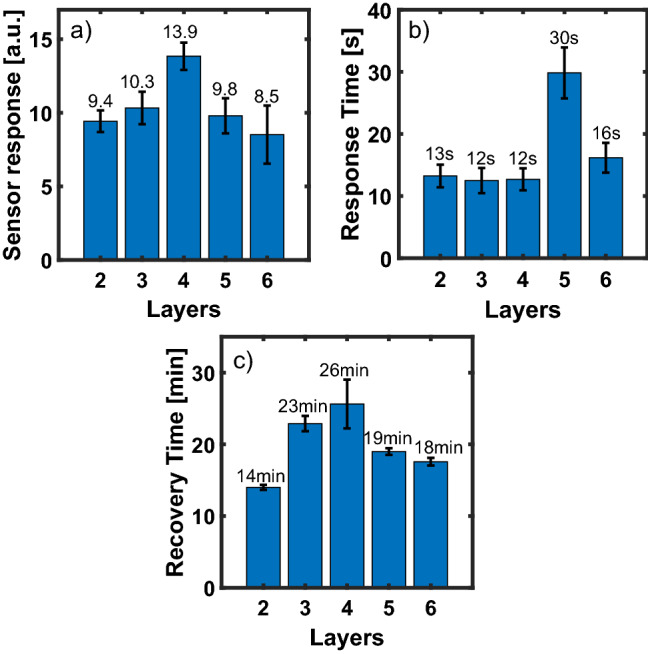
(**a**) Mean values ± Standard deviation (SD) of sensor response (**a**), response time (**b**) and recovery time (**c**), using devices with different number of PbS QDs layers, taken after 100 s of 30 ppm NO_2_ exposure at room temperature.

A systematic analysis of the effect of the film thickness on the NO_2_ gas sensing was then carried out for a fixed target gas concentration of 30 ppm. Figure 7a shows the results obtained using devices with a number of PbS QDs layers ranging from 2 to 6. For each thickness, we reported the mean and the standard deviation of gas response estimated using the resistance data of several sensors during 8 cycles of exposure to NO_2_.

The best performance in terms of gas response is obtained with a 4-layers device with a mean value of 13.9 Ω/Ω, representing a significant improvement with respect to both thinner and thicker devices.

Figure 7b and c show the corresponding response and recovery time at room temperature. As expected, the recovery time shows a bell-shaped trend consistent with that of the gas response, while no dependence of the response time on the number of layers is observed.

We also observed a direct proportionality between recovery time and gas concentration, with shorter times at low ppm of NO_2_. Compared to other NO_2_ sensing devices, our sensors show a slow recovery time; this aspect could be improved operating the sensors at higher temperatures or changing the surface characteristics of the QDs (either changing their size or employing specific ligands) at the cost of a lower sensitivity. However, due to the hazard represented by NO_2_ in air, NO_2_ sensors are expected to give a fast alarm in case of dangerous gas concentration, but do not necessarily require very fast recovery.

The relationship between the CQDs film thickness and the sensor response is non-trivial, but we could clearly identify an optimal thickness for NO_2_ detection. We can tentatively attribute the sensor response dependence on the film thickness to a combination of chemical and electrical mechanisms.

Gas molecules reach the top surface of the sensor and react with the QDs. Depending on the gas concentration, some gas molecules should also diffuse towards the bottom of the film and react with the deeper layers. When reacting with the QDs, the gas molecules act as doping species, enhancing the free carrier concentration. At the same time, current is forced through the QDs film by the two metal contacts. Due to the morphology of the film and of the metal contacts, the electric field may be non-uniform inside the sensor. If the film is too thick, the QDs reacting with the gas are located in the upper layers, i.e. in a lower electric field area, whereas deeper QDs layers do not interact with the analyte gas; on the other hand, if the film is too thin, the number of QDs available for interaction with the gas molecules is lower and the resulting sensor response is reduced.

The theoretical detection limit for a 4-layers device was estimated with a linear fit of the sensor response versus the NO_2_ concentration in the 2.8–10 ppm range as shown in Fig. 6b. The response slope of the linearized characteristic was 0.91 ppm^−1^ with a correlation coefficient of 0.998, indicating a good linearity of the experimental data in the low concentration range.

The sensor noise was evaluated using the standard deviation of the measured sensor resistance before the NO_2_ exposure over 400 data points acquired in an 80 s timespan. The theoretical detection limit of the sensor, with a signal-to-noise ratio (SNR) of 3, was estimated to be approximately 0.15 ppb at room temperature according to Eq. (5)^38^:

5 $$DL = 3\frac{{RMS_{noise} }}{Slope}$$

The detection limit has been evaluated following the same procedure also for 2-layers and 8-layers devices, obtaining 1 ppb and 0.55 ppb, respectively. As expected, these results confirm the 4-layers device higher performance. Finally, we compared the results obtained with our 4-layers devices with state-of-the-art PbS-based NO_2_ sensors previously reported in literature.

Due to limitations of the measurement setup, we could not characterize the device response for gas concentrations lower than 2 ppm; thus, in the evaluation of the detection limit, we assumed a linear sensor response below the lowest measured concentration, as previously reported for similar QD-based gas sensors^20,41^.

Even if our measurement setup did not allow for device characterization at NO_2_ concentration lower than 2.8 ppm, our results demonstrate the good performance of the devices for their employment in safety applications. Typical NO_2_ alert levels in industrial workplaces are, in fact, in the 5 ppm range, as suggested by the Occupational Safety and Health Administration of the United States Department of Labor (OSHA). The calculated detection limit suggests that the proposed sensors could be also employed for environmental and pollution monitoring, where sub-ppm NO_2_ concentrations need to be measured. For these specific applications, however, further measurements would be needed to confirm the evaluated detection limit.

Table 1 reports the sensor response, rise time, recovery time and detection limit of several NO_2_ sensors. In terms of sensor response and response time, our device’s performance is similar to previously published results; nevertheless, the proposed device shows a significant improvement in the detection limit with respect to state-of-the-art sensors. This enhancement can be attributed to a reduced current noise.

**Table 1:** Recent room temperature NO_2_ sensors using PbS as sensing material.

Sensing material	Substrate	Method	Temperature	Detection range	Detection limit	Best response	T90/T10	References
PbS film	Glass	Chemical bath deposition	38 °C	5–100 ppm	–	3.85 (100 ppm)	20 s/1477 s (100 ppm)	Navale 2015^39^
PbS CQDs	VHB	Spin-coating	RT	5–100 ppm	13 ppb	125 (50 ppm)	7 s/22 s (50 ppm)	Song 2018^40^
PbS CQDs	Paper	Spin-coating	RT	0.5–50 ppm	84 ppb	21.7 (50 ppm)	12 s/37 s (50 ppm)	Liu 2014^20^
PbS CQDs	Al2O3	Spray-coating	RT	2–100 ppm	56 ppb	44 (50 ppm)	1 s/28 s (50 ppm)	Min Li 2016^41^
PbS CQDs	SiO2/Si	Drop-casting	RT	2.8–100 ppm	0.15 ppb	21 (30 ppm)	5 s/1560 s (30 ppm)	This work

## Conclusions

In this work, we developed high-performance chemiresistive PbS CQDs sensors for room temperature NO_2_ detection. Devices were fabricated by drop casting PbS CQDs onto interdigitated metal contacts on SiO_2_/Si chips. The long-chained organic ligands removal ensured excellent accessibility by gas molecules to the QDs surface and provided suitable transport properties and stability. The device was able to detect NO_2_ selectively among other air pollutants such as CH_4_, CO and CO_2_ and it was successfully tested towards different gas concentrations in the 2.8–100 ppm range. It was demonstrated that the gas sensing characteristics of PbS QDs films are influenced and may be enhanced significantly by the control of the film thickness: the highest response was observed for the 4-layers device. For such a device, a detection limit of about 0.15 ppb has been estimated from the slope of the sensor response and its noise. Compared to state-of-the-art NO_2_ sensors based on PbS, the proposed device shows a significant improvement in the detection limit.

In conclusion, the gas sensor based on PbS CQDs exhibited excellent sensitivity to NO_2_, rapid-response and full recovery after gas release.
